# Common Wheat Pasta Enriched with Ultrafine Ground Oat Husk: Physicochemical and Sensory Properties

**DOI:** 10.3390/molecules28207197

**Published:** 2023-10-20

**Authors:** Beata Biernacka, Dariusz Dziki, Renata Różyło, Urszula Gawlik-Dziki, Renata Nowak, Wioleta Pietrzak

**Affiliations:** 1 Department of Thermal Technology and Food Process Engineering, University of Life Sciences in Lublin, 31 Głęboka St., 20-612 Lublin, Poland; beata.biernacka@up.lublin.pl; 2 Department of Food Engineering and Machines, University of Life Sciences in Lublin, 28 Głęboka St., 20-612 Lublin, Poland; renata.rozylo@up.lublin.pl; 3 Department of Biochemistry and Food Chemistry, University of Life Sciences in Lublin, 8 Skromna St., 20-704 Lublin, Poland; urszula.gawlik@up.lublin.pl; 4 Department of Pharmaceutical Botany, Medical University of Lublin, 1 Chodźki St., 20-835 Lublin, Poland; renata.nowak@umlub.pl (R.N.);

**Keywords:** pasta, micronized oat husk, hull, quality, texture, antioxidant properties, pheonolic compounds profile

## Abstract

Oat husk (hull) is a byproduct of oat processing that is rich in insoluble fiber. The aim of the study was to evaluate the effect of partially replacing wheat flour with oat husk (at levels of 0, 5, 10, 15, and 20 g/100 g) on the physicochemical properties and sensory acceptance of pasta. Additionally, UPLC-MS/MS analysis was performed to identify phenolic acids and flavonoid compounds, and the cooking properties of the pasta were evaluated. The test results indicate that oat husk significantly (*p* < 0.05) increased the ash and fiber contents in the pasta, while decreasing the protein and fat contents. Moreover, the redness and yellowness of both raw and cooked pasta increased, while lightness decreased as a result of pasta enrichment with oat husk. Oat husk also led to a decrease in the stretching force of cooked samples, although cooking loss increased significantly but did not exceed 8%. The contents of phenolics and antioxidant activity significantly increased with the incorporation of hull in pasta recipes. UPLC-MS/MS analysis showed that the enriched pasta was especially abundant in ferulic acid. Products with up to 10 g of husk/100 g of wheat flour showed good consumer acceptance. However, higher levels of this additive led to notably lower assessments, particularly in terms of pasta texture.

## 1. Introduction

In recent years, there has been noticeable progress in research on dietary fiber (DF) and legislative work related to healthy eating guidelines. Based on current knowledge of DF’s impact on human health, doctors and nutrition experts have developed recommendations for the minimum daily intake of this essential food ingredient. Across various countries, the suggested amount ranges from 18 to 38 g/day for an adult [1]. According to guidelines from the FAO, WHO, EFSA, and the US Food and Drug Administration (US FDA), the recommended daily intake of DF is set at 25 g/day [1,2].

According to the latest data, cereal products serve as the basic source of DF in daily diets. To maintain the leading position of cereal products, it would be desirable to develop new and sensorially attractive whole grain products, as well as products enriched with high-fiber additives derived from the outer layers of the grain [3]. 

All low-processed cereal products derived from whole grains and their peripheral layers are significantly richer in DF compared to their highly processed products [4,5]. Oats, used as a raw material in the food industry, have a unique chemical composition that makes them highly beneficial for both healthy and ill individuals [6]. Products based on oats contain essential nutrients, including macronutrients (proteins, lipids, and carbohydrates) and micronutrients (mineral salts and vitamins) [7]. Notably, the soluble DF in oats contributes to lowering the glycemic index [8,9].

Oat grains are rich in valuable exogenous amino acids and primarily consist of unsaturated fatty acids (EFAs)—essential fats that must be obtained through the diet [10,11]. These include oleic, linoleic, and α-linolenic acids, along with longer-chain fatty acids such as eicosapentaenoic, docosahexaenoic, and arachidonic acids. Additionally, oats are an excellent source of essential minerals such as iron, magnesium, manganese, calcium, copper, potassium, zinc, phosphorus, and silicon. They also contain significant levels of vitamin E, a potent antioxidant, as well as pantothenic acid and thiamine [7,10,11,12]. Oat and oat byproducts also serve as carriers for fat-soluble vitamins A, D, E, and K. Furthermore, they contain bioactive compounds with documented health benefits. These include antioxidants like phytic acid and polyphenolic compounds (such as avenanthramides, ferulic acid, and sinapic acid), as well as substances such as unsaturated fatty acids (EFAs), β-glucans, α-linolenic acid, and phytosterols [10,13]. 

Incorporating oat and oat byproducts into food formulations can not only improve the nutritional quality of the products, but also appeal to health-conscious consumers seeking natural and beneficial ingredients in their diets. Moreover, oat byproducts are an excellent source of soluble fiber and aid in maintaining a healthy digestive system by preventing constipation and promoting regular bowel movements [6]. Additionally, oat byproducts can be used to improve the texture and flavor of functional foods [10]. To enhance the nutritional value and improve the quality of cereal food, various additives are used in production. One such interesting additive is micronized oat husk (MOH), a by-product of oat processing. MOH is a rich source of insoluble DF [4], as well as bioactive compounds such as phenolic acids and flavonoids [5]. While it is primarily used in animal feed, MOH can also be used as an ingredient in high-fiber foods [6,7]. Limited research exists that explores the potential of using MOH as a food additive. MOH can be an interesting additive for common wheat pasta. Natural oat fiber, specifically micronized oat fiber [8], not only positively impacts the digestive system, but also has broader effects on the whole body. In fiber-rich plant materials, micronization enhances water absorption by particles and increases fiber solubility [9]. It also improves mouthfeel and increases the release of flavors [8]. Importantly, micronization improves the extraction of antioxidant compounds and helps in the release of many bioactive compounds associated with the food matrix [10].

The aim of this study was to enrich common wheat pasta with MOH and to evaluate the quality and physicochemical properties of the resulting enriched products. Both the sensory properties of the pasta and its culinary characteristics were determined. The chemical composition of the products, as well as texture, color, antioxidant properties, and the profile of phenolic compounds, were also assessed.

## 2. Results and Discussion 

### 2.1. Basic Composition of Raw Materials and Pasta

The chemical composition analysis of the raw materials showed that MOH had the highest ash and fiber content, at 3.42% and 90.7%, respectively, while CWF had the highest protein content at 11.95% (Table 1). The enrichment of MOH into pasta led to minor changes in dry matter and water activity. The dry matter values ranged from 90.46% in the control sample to 92.25% in the pasta with the highest level of MOH addition (P20). It can be concluded that dry matter increases as MOH content increases (*r* = 0.95, *p* < 0.05). The water activity in the pasta samples ranged between 0.302 and 0.352 (for P20 and P5, respectively); in all tested products, the water activity levels were sufficient to ensure microbiological stability. 

The addition of MOH significantly increased the fiber content, ranging from 0.27% to 7.05%, and the ash content, ranging from 0.36% to 1.41%. Conversely, the fat and protein contents decreased from 1.26% to 0.96% and from 11.89% to 10.34%, respectively. The content of available carbohydrates in the pasta also decreased with the addition of MOH, from 86.22% (in the control sample, P0) to 80.24% (in the sample with the highest MOH addition, P20). Many studies have confirmed that the addition of the soluble oat fiber fraction (β-glucan) to cereal products increases the β-glucan content in the final product. For example, incorporating 80 g/kg of oat fiber powder increased the TDF content to a level that would qualify the pasta for a “high in fiber” nutritional claim [11]. Other studies have also shown that partially replacing wheat flour with oat bran led to an increase in fiber and ash content, along with a decrease in the available carbohydrates, in pasta [14,15]. A similar trend was observed in bread production, where wheat flour was supplemented with different amounts of oat flour. This mixture exhibited the highest content of DF, particularly β-glucans, which are considered to be a prebiotic fiber [12].

### 2.2. Color Parameters of Pasta

CWF was lighter than MOH, with L* values of 90.76 and 74.51, respectively (Table 2). On the other hand, MOH was more red and yellow. As the amount of MOH in the recipe increased, the lightness of the uncooked pasta decreased, ranging from 66.48 (in the control sample) to 48.66 (in pasta with 20 g/100 g MOH). Based on the measurement of lightness, it was found that raw pasta samples enriched with MOH were darker than common wheat pasta (P0). The redness of raw pasta increased from 3.27 (P0) to 6.09 (P20) due to MOH addition. Similarly, the yellowness of uncooked pasta increased with the addition of MOH, as indicated by the increase in the b* value from 13.63 for P0 to 16.67 for P20. The total color difference (Δ*E*) between the control and enriched pasta ranged from 4.70 to 18.02, and a significant correlation between MOH content and Δ*E* was observed (*r* = 0.99, *p* < 0.05).

Importantly, cooking the pasta enhanced the color differences between the control and enriched products. The Δ*E* values for the cooked MOH-enriched pasta ranged from 5.34 to 16.96, and a significant correlation between MOH content and ΔE was also observed (*r* = 0.98, *p* < 0.05). A color difference above three is already recognizable to the observer, so even a modest addition of 5 g/100 g MOH significantly affected the color changes. Cooking led to a noticeable decrease in the L* value for most pasta samples. The lightness of the cooked samples ranged from 67.26 for CP0 to 53.17 for CP20. Moreover, redness (a*) increased with the addition of MOH to the pasta. The highest a* value was observed for the CP20 sample (9.62), while the lowest was for CP0 (2.02). A significant correlation between MOH content and a* was also found (*r* = 0.96, *p* < 0.05). Regarding the aspect of yellowness, cooked pasta enriched with MOH showed significantly higher b* values compared to the control sample. The yellowness value increased from 16.51 for CP0 to 22.04 for CP20. 

In summary, it was observed that the addition of MOH significantly affected the color of the pasta, both before and after cooking. This finding is consistent with what has been reported for the addition of fiber to other cereal products made from wheat flour. For instance, it was proven that incorporating up to 5 g/100 g of carob fiber into CWF had a strong impact on pasta color, particularly leading to a decrease in lightness and redness. Additionally, cooking increased the lightness and redness of pasta containing carob fiber, but decreased its yellowness [13]. Piwińska et al. showed that the addition of oat β-glucan fiber affected the color of durum wheat pasta. Pasta made with semolina substitution was darker compared to control samples [11]. Other authors found that the addition of MOH notably enhanced the redness of shortbread cookies, while slightly reducing yellowness. However, significant changes were observed when 10% or more of MOH was added [6].

### 2.3. Cooking Properties of Pasta

Ideally, pasta should retain its shape and increase in volume, with minimal material loss, during cooking [16]. The research showed that the optimal cooking time (OCT) for pasta increased with the addition of MOH, rising from 10.50 min for CP0 to 12.60 min for pasta containing 20 g MOH/100 g flour (Table 3). A significant and positive correlation between cooking time and MOH addition was observed (*r* = 0.99, *p* < 0.05). Moreover, the inclusion of MOH led to a significant decrease in the weight increase index (WI), from 2.28 (CP0) to 2.10 (CP20). MOH also notably increased cooking loss, which ranged from 4.62% for CP0 to 7.52% for CP20 (*r* = 0.87, *p* < 0.05). Other authors [11,15] showed that the incorporation of soluble fiber can lead to alterations in the pasta structure, subsequently affecting the rate at which starch is broken down. In particular, the inclusion of oat β-glucan fiber powder affected the cooking quality of durum wheat pasta by increasing its swelling index, water uptake, and cooking loss (CL) [11]. 

### 2.4. Texture Properties

The data from the research indicate that the addition of MOH to pasta significantly influenced its mechanical properties, specifically its deformation and stretching force (Table 4). As the amount of MOH in the recipe increased, both deformation and stretching force decreased. Statistically significant correlations were found between the amounts of MOH and both deformation and stretching force (r = −0.99, *p* < 0.05 and *r* = −0.86, *p* < 0.05, respectively). These findings corroborate similar trends reported by other authors, who studied the incorporation of carob fiber into common wheat pasta [13]. According to Piwińska et al., a decrease in hardness and adhesiveness was observed when oat-soluble fiber powder was added to the pasta [11]. Other tests on pasta samples made with 50/50 durum wheat semolina/oat bran showed an increase in optimal cooking time and cooking loss [15]. In terms of cooking time, the highest values of deformation and stretching force were seen for pasta with the shortest OCT. Significant and positive correlations were also noted between cooking time and both deformation and stretching force (*r* = 0.98, *p* < 0.05 and *r* = 0.86, *p* < 0.05, respectively).

### 2.5. Total Phenolic Content and Antioxidant Activity of Cooked Pasta

Increasing evidence suggests that polyphenols offer multiple benefits to humans, including potent antioxidant properties, prevention of diseases induced by oxidative stress, and particularly, protection against cardiovascular diseases, neurodegenerative diseases, and cancers [17,18]. Table 5 displays the content of polyphenols and antioxidant activity (AA) in both raw materials and pasta samples. The total phenolic content (TPC) in CWF was nearly 2.5 times lower than that in MOH. Additionally, AA against ABTS and DPPH was significantly higher in CF compared to MOH, and exhibited different EC_50_ values. MOH demonstrated a high capacity for reducing and neutralizing free radicals; specifically, the EC_50_ values for ABTS and DPPH were 35.62 and 55.22 mg d.m./mL, respectively (the higher the EC_50_, the lower the reduction ability and antioxidant properties). Significant differences in TPC were observed between the pasta samples tested and the MOH fiber-enriched pasta. Notably, a substantial and statistically significant increase in TPC content was evident. For instance, the TPC for the CP0 sample was 247.59 mg GEA/g d.m., while for the CP20 sample, an increase of over 41% was recorded, yielding 350.20 mg GEA/g d.m. A significant correlation was found between MOH and TPC (*r* = 0.94, *p* < 0.05). Moreover, MOH enhanced the AA of MOH-enriched pasta compared to the control sample. Significant differences in AA became apparent when MOH was added at a concentration of 5 g/100 g for both ABTS and DPPH. As the quantity of MOH in the pasta recipe increased, so too did the AA of the cooked pasta (indicated by decreasing EC_50_ values). In the case of ABTS, the EC_50_ value significantly decreased upon the addition of MOH from 256.53 mg d.m./mL (control sample) to 101.17 mg d.m./mL (CP20). A similar trend was observed for DPPH: MOH elevated the pasta’s ability to neutralize DPPH free radicals (indicated by a decreasing EC_50_ value). Specifically, the EC_50_ values of DPPH for CP0 and CP20 were 364.04 mg d.m./mL and 162.02 mg d.m./mL, respectively. Statistically significant correlations were found between GL and antioxidant activities (*r* = 0.97 and 0.99 for ABTS and DPPH, respectively). These increases in AA are attributed to the bioactive compounds present in oats seeds [19]. Previous research has shown that MOH is a valuable source of biologically active compounds, especially phenolic acids, which exhibit strong antioxidant properties [9,20,21]. Specifically, MOH is a rich source of phenolic acids, including ferulic (the dominant one), caffeic, p-hydroxybenzoic, vanillic, syringic, and sinapic acids [9]. The results also reveal a significant correlation between TPC and both ABTS (*r* = 0.89, *p* < 0.05) and DPPH (*r* = 0.91, *p* < 0.05). Comparable findings have been reported by other authors, showing that the partial replacement of wheat flour with micronized OH led to increased AA in shortbread [6], as well as in pasta [11]. Research has shown that, compared with the control pasta, the pastas with added oat bran showed increased antioxidant capacity [15]. Levent et al. showed that the total phenolic and antioxidant activities of pasta increased significantly after oat bran addition [14]. Therefore, MOH powder can be effectively used in nutritionally valuable pasta recipes.

### 2.6. LC-ESI-MS/MS Analysis of Phenolic Acids and Flavonoid Compounds

UPLC-MS/MS analysis revealed the presence of phenolic acids, as well as flavonoid aglycones and glycosides, in the pasta samples enriched with MOH. Trace amounts of flavonoid aglycones were identified in all the tested samples (Table 6). Regarding flavonoid glycosides, trace amounts of luteolin-7-*O*-glucoside (luteoloside) were found in the control sample, but were absent in other samples. In addition, among the flavonoid glycosides, trace amounts of apigenin-6-C-glucoside (Isovitexin)/apigenin-8-C-glucoside (apigenin-6-C-glucoside (Vitexin)) were detected in the P0, P5, and P10 samples. In contrast, in the P15 and P20 samples, this flavonoid was present in more significant amounts, registering 19.75 and 28.40 µg/g d.m., respectively. 

The UPLC-MS/MS analysis identified eight phenolic acids in the pasta samples enriched with MOH, as outlined in Table 7. Three of these acids were either in trace amounts or not detected in all samples. Conversely, three specific acids—5-caffeoylquinic acid (chlorogenic acid), vanillic acid, and syringic acid—were present in pasta samples where MOH was added at concentrations exceeding 15 g/100 g. The presence of 4-hydroxycinnamic acid (also known as p-coumaric acid) was noted even in samples with just a 5 g/100 g MOH addition. Additionally, the content of p-coumaric acid increased in proportion to MOH addition, measuring 54 µg/g d.m. for P5 and 292 µg/g d.m. for P20, respectively. A growing number of studies have shown several bioactivities of coumarins and coumarin derivatives, including free radical scavenging, anticancer, anti-inflammatory, antimicrobial, and antithrombotic effects [22,23,24]. These beneficial properties make them important starting products for the development of new therapeutic agents [18]. Ferulic acid was the dominant phenolic acid, and was found in each sample. Its presence increased with the addition of MOH, averaging 1187.30 µg/g d.m. Ferulic acid is known for its low toxicity and a wide range of physiological functions, such as anti-inflammatory, antioxidant, antimicrobial, anticancer, and antidiabetic activities [10]. Consequently, it has found applications across multiple industries, including pharmaceuticals, food, and cosmetics [18]. This acid not only acts as a free radical scavenger, but also inhibits enzymes that catalyze the generation of free radicals while enhancing the activity of scavenger enzymes [9]. 

### 2.7. Sensory Properties

**Figure 1:**
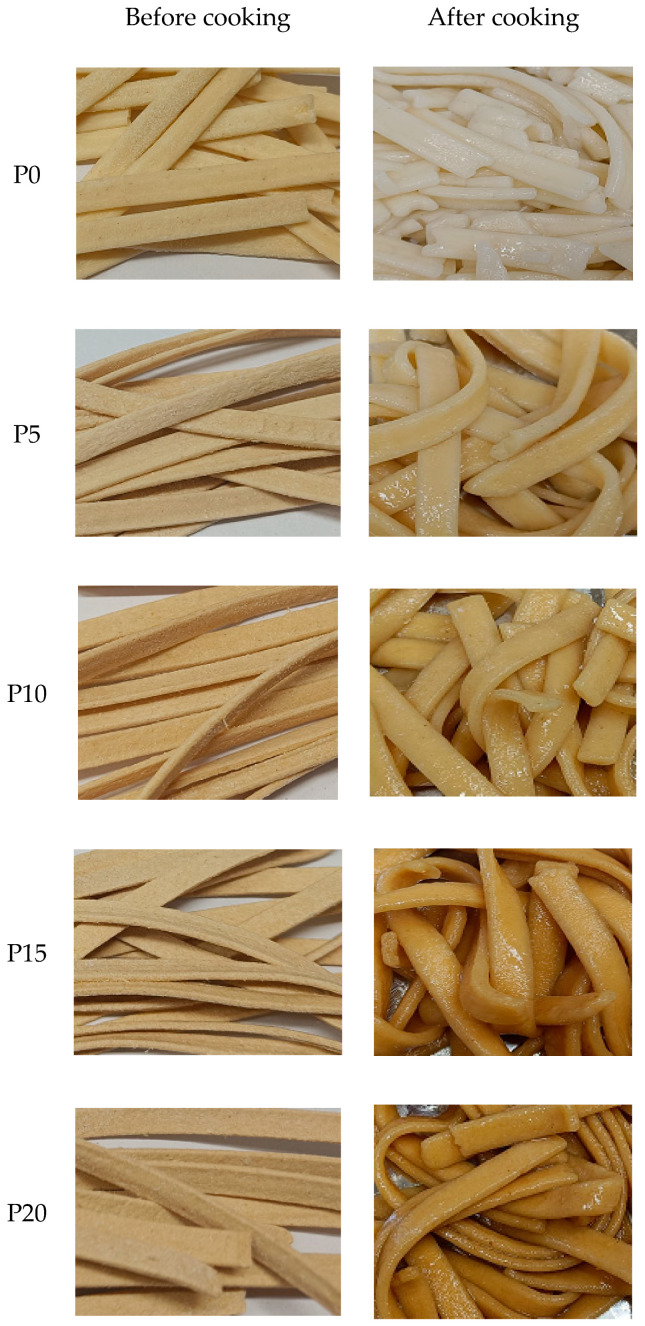
Pasta before and after cooking with the addition of MOH, P0, P5, P10, P15 and P20—pasta with 0, 5, 10, 15 and 20 g/100 g of micronized oat husk, respectively.

Pasta samples enriched with 5–20 g/100 g of MOH before and after cooking are shown in Figure 1.

The addition of MOH up to 10 g/100 g did not negatively impact the overall assessment of the tested characteristics (Table 8). In terms of appearance, CP10 and CP20 received the highest ratings (6.33), while CP15 was rated slightly lower (6.00). MOH also affected the color of pasta, which ranged from light grey to slightly brown, but it was solid. The CP15 and CP20 samples were considered to have the best color (6.00), whereas CP0 was judged to have the least appealing color (2.33). Regarding aroma, CP0 received the highest rating (6.33) for its distinct, pleasant smell, while CP5 was rated slightly lower (6.00). Pasta with the addition of MOH above 10 g/100 g in terms of smell was rated at the same level (5.67), with a slightly perceptible smell of oat husk. In terms of taste, CP0 and CP5 rated higher (5.67), followed by CP15 (5.33); CP20 had the lowest taste rating (4.00). MOH also adversely affected pasta texture, with CP20 receiving the lowest texture rating (3.67). Texture is a complex sensory property influenced by multiple senses. Higher MOH levels (>5 g/100 g) led to diminished texture attributes. In summary, CP10 and CP5 had the highest scores for overall quality (6.33 and 6.00, respectively), while CP15 was rated slightly lower (5.00) in terms of overall acceptability. CP20 had lower scores in both taste and texture, resulting in the lowest overall rating. Sensory evaluation indicated that pasta enriched with up to 10 g/100 g of MOH was generally well-accepted. The study of Piwińska et al. [11] showed that pasta with oat β-glucan fiber powder was darker, softer, and less sticky than semolina pasta [11]. Other authors have found that substituting wheat flour with oat husk in amounts ranging from 5 to 20% did not significantly affect the overall acceptability of shortbread cookies [6]. Current research has shown that the addition of oat fiber to pasta affects its sensory characteristics, while our previous study demonstrated a positive influence on the sensory attributes and cooking quality of pasta enriched with carob fiber [13].

## 3. Materials and Methods

### 3.1. Basic Composition of Raw Materials

In this study, CWF (type 500, with a granulation range of 200–300 μm) provided by Lubella (Lublin, Poland) and MOH produced by (Gdynia, Poland) were used. The preparation of the enriched pasta followed the methodology outlined by Dziki et al. [6]. All chemicals used in the study were of analytical grade. The cost of using MOH in pasta production is comparable to using semolina. Therefore, producing pasta from common wheat flour with the addition of MOH will not significantly impact the price of pasta.

### 3.2. Research Materials and Preparation of Pasta

**Figure 2:**
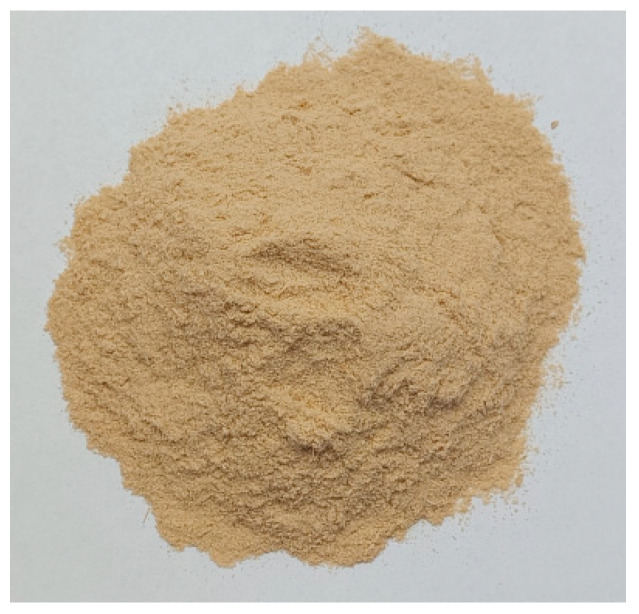
Micronized oat husk.

The pasta was made using CWF, which was replaced with MOH (Figure 2) at levels of 0, 5, 10, 15, and 20 g/100 g. The pasta-making process and parameters adhered to the procedure described in Biernacka et al. [25]. To start the dough preparation, the ingredients were measured and sifted to remove any lumps. All ingredients were then placed in the bowl of a KitchenAid planetary food processor, model T-5KPM5EER (Greenville, SC, USA). Because fiber absorbs water readily, the amount of water added to the dough had to be adjusted with increasing MOH levels. Specifically, 48.3, 51.7, 53.3, 56.7, and 60.0 g of water/100 g of CWF with MOH was used for P0, P5, P10, P15, and P20, respectively. The device formed the dough with the help of the rolling and cutting attachment (KitchenAid 5KSMPSA, Greenville, SC, USA). The tagliatelle had approximate dimensions of 2 mm in thickness and 5 mm in width. The prepared pasta was then hung on a KitchenAid 5KPDR pasta dryer (Benton Harbor, MI, USA) and placed in a climatic chamber (ICH 256, Düsseldorf, Germany) for 24 h at a temperature of 25 °C and 20% relative humidity. The drying process continued until the moisture content of the pasta reached between 11% and 12% wb. Drying the pasta took about 24 h. The pasta was stored in tightly closed polyethylene bags, without access to light and oxygen. The storage time is similar to that of commercial pasta, and equals a maximum 3 months.

### 3.3. Evaluation of Chemical and Physical Properties of the Pasta 

#### 3.3.1. Determination of the Basic Chemical Composition 

CWF, MOH, uncooked control pasta (P0), and pasta with MOH were subjected to chemical analysis to determine the contents of dry matter, crude protein, crude ash, and crude fat. The analyses followed Method 44-15A for dry matter, Method 08-01 for crude protein, Method 46-06 for crude ash, and Method 30-10 for crude fat. The fiber content was tested using the Method 993.21 (AOAC 2011) guidelines. Available carbohydrates were calculated based on differences in the contents of dry matter, protein, fat, ash, and fiber. All analyses were carried out in triplicate and with adherence to AOAC standards [26]. 

#### 3.3.2. Measurement of Water Activity 

The measurement of water activity in pasta was carried out at 20 °C using a LabMaster device (Novasina AG, CH-8853 Lachen, Switzerland), following the procedure described by Serin, Turhan and Turhan [27]. A 2 g sample of dry pasta was placed in the measuring vessel for the analysis.

#### 3.3.3. Color Coordinates 

The color coordinates of raw materials, as well as uncooked and cooked control pasta, were determined using the CIEL*a*b* system with a colorimeter (CR-400C Chroma Metre 115, Minolta, Color Lab, Osaka, Japan). In the CIEL*a*b* system, L* represents lightness, ranging from 0 (perfect black body) to 100 (perfect white body). The a* coordinate indicates the shift from green color (−a*) to red color (a*), while b* signifies the color transition from blue (−b*) to yellow (b*) [26,28]. Changes in color due to the addition of GL powder were assessed by calculating the color differential index (Δ*E*) by the methodology described by Monteiro et al. [29].

### 3.4. Cooking Quality of Pasta 

#### 3.4.1. Optimal Cooking Time 

OCT was determined according to the AACC 66–50 [30]. OTC is the cooking time after which the white core of the pasta disappears when the cooked pasta is squeezed between a pair of glass plates.

#### 3.4.2. Weight Increase Index and Cooking Loss

WI is the weight of pasta after cooking compared to the weight of pasta before cooking. CL this is the amount of substances penetrating the water during the cooking of pasta, expressed in %. WI and CL were calculated according to the methods described by Bonomi et al. [31] and Biernacka et al. [32], respectively.

### 3.5. Texture Analysis

Texture analysis was performed on a Zwick testing machine (Zwick GmbH and Co., Ulm, Germany). Cooked pasta (Section 3.4.2) underwent a stretching test following the procedures outlined by Wójtowicz [33]. For measuring pasta elongation, a Tensile Kiefer Dough and Gluten Extensibility Rig on an Instron 5564 with a 50N head (Stable Micro Systems Ltd., Godalming, UK) was utilized. The tensile test speed was set at 3.3 mm/s. A single sample of cooked pasta was placed on the test table and covered with plastic during the test. Throughout the tensile tests, the stretching force was assessed using a computer program. The values in the curves represent the averages of five replicates.

### 3.6. Total Phenolic Content and Antioxidant Activity

For extract preparation, a 0.5 g sample of ground dried material was extracted with 50% methanol, and shaken three times for 30 min. The homogenate was then centrifuged at 4000 rpm for 10 min at a temperature of 4 °C [34]. This extraction process was performed twice. The obtained supernatants were combined and used for further analyses. 

TPC was determined using the Folin–Ciocalteu method [35]. The TPC values are expressed as Gallic acid equivalents (GAE) per gram of dry weight.

The AA extracts were assessed via the following analyses:

- The ability to scavenge ABTS (2,20-azinobis (3-ethylbenzothiazoline-6-sulfonate) free radicals, as per the method outlined by Re et al. [36];
- The ability to neutralize DPPH (2,2-diphenyl-1-picrylhydrazyl) free radicals, according to the procedure described by Brand-Williams et al. [37].

According to Singh et al. (2020), the strength of a compound is inversely related to its EC_50_ value. In other words, a lower EC_50_ value indicates a stronger compound. Consequently, the compound with the lowest EC_50_ value is considered the strongest [38].

### 3.7. LC-ESI-MS/MS Analysis of Phenolic Acids and Flavonoid Compounds

The contents of phenolic and flavonoid compounds were analyzed using high-performance liquid chromatography coupled with electrospray ionization mass spectrometry (LC-ESI-MS/MS). This employed a slightly modified method previously described by Nowacka et al. [39] and Pietrzak et al. [40]. An Agilent 1200 Series HPLC system (Agilent Technologies, Santa Clara, CA, USA) was connected to a 3200 QTRAP Mass spectrometer (AB Sciex, Framingham, MA, USA). The electrospray ionization source (ESI) operated in negative-ion mode and was used for all analytes. Both systems were controlled by Analyst 1.5 software (AB Sciex, USA), which was also used for data interpretation. 

The separation of phenolic and flavonoid compounds was carried out at 25 °C using a Zorbax SB-C18 column (2.1 × 100 mm, 1.8 mm particle size; Agilent Technologies, USA). The mobile phase consisted of 0.1% aqueous formic acid (solvent A) and acetonitrile with 0.1% formic acid (solvent B). The injection volume was set at 3 µL, and the flow rate was 300 µL/min. The gradient was programmed as follows: 0–2 min—20% B; 3–4.5 min—25% B; 5.5–7 min—35% B; 8–12 min—65% B; 14–16 min—80% B. The total run time was 28 min. 

The ESI-MS worked in negative ion mode, with parameters set as follows: capillary temperature at 550 °C, curtain gas pressure at 30 psi, nebulizer gas pressure at 50 psi, and source voltage at −4500 V.

Triplicate injections were performed for each standard solution and sample. The limits of detection and quantification for all analytes were determined based on signal-to-noise ratios of 3:1 and 10:1, respectively. The qualitative identification of compounds was achieved by comparing MS/MS spectra and LC retention times with corresponding standards tested under identical conditions. Calibration curves obtained in MRM mode were used for the quantification of all analytes.

### 3.8. Sensory Evaluation

Cooked pasta was evaluated for its appearance, color, smell, taste, and texture. These features were further detailed in terms of firmness and adhesiveness, leading to an overall assessment. The evaluation panel consisted of 63 untrained consumers, aged 24–45, including 22 women and 21 men. A hedonic scale was valued to assess each feature, ranging from 1 point (least acceptable) to 7 points (most acceptable). During the evaluation of organoleptic characteristics, adequate lighting was ensured, and the room was protected against foreign odors [31]. 

### 3.9. Statistical Estimation of Research Results

Pasta was produced in three repetitions. For the research, samples of pasta from all repetitions were mixed, and then they were randomly selected for analysis. The independent variable was the proportion of oat flour in wheat flour. The dependent variables included all evaluated physicochemical and sensory properties of the pasta. Experimental data were analyzed using one-way Analysis of Variance (ANOVA) with the aid of STATISTICA 6 software (StatSoft, Inc., Tulsa, OK, USA). Tukey’s test was used to determine the significance of differences between the means. All measurements, except for texture analysis, were conducted in triplicate. The stretching test was performed with five replicates. Additionally, Pearson’s correlation coefficients were calculated. All tests were performed at a significance level of α = 0.05.

## 4. Conclusions

In this study, we have demonstrated that micronized oat bran can be used as a valuable functional additive in pasta production. The enrichment of MOH into pasta led to a slight decrease in protein and fat content, while elevating the levels of ash and fiber. MOH significantly influenced the color of both the raw and cooked pasta, particularly by reducing the lightness while enhancing redness and yellowness. Additionally, MOH affected the cooking properties of the pasta: OCT and CL increased, whereas WI decreased. Notably, adding MOH to wheat flour significantly reduced the deformation and stretching force of the cooked product. MOH also significantly boosted the TPC and enhanced the antioxidant capacity of the pasta. During sensory evaluation, a higher MOH content positively affected the pasta’s appearance and color, although it adversely affected its texture. Most importantly, it was found that the addition of MOH (up to 10 g/100 g) was well-received by consumers and provided a valuable source of fiber and bioactive compounds, particularly ferulic acid. The obtained results may serve as fundamental guidelines for the production of pasta from common wheat flour enriched with MOH. This additive is an inexpensive raw material that will not have a negative impact on the final price of the pasta. In future studies, it will be verified under industrial conditions. Moreover, future studies should focus on the potential utilization of MOH as a functional additive for enriching durum wheat pasta. 

## Figures and Tables

**Table 1:** Chemical composition (% dry matter) and water activity of the pasta enriched with MOH.

Sample	Parameter						
	Dry Matter (%)	Ash (%)	Protein (%)	Fat (%)	Fiber (%)	Water Activity (-)	Available Carbohydrates (%)
CWF *	86.15 ± 0.35 ^A^**	0.39 ± 0.12 ^A^	11.95 ± 0.09 ^A^	1.20 ± 0.12 ^A^	3.20 ± 0.63 ^B^	0.393 ± 0.054 ^B^	83.26
MOH	89.80 ± 0.11 ^A^	3.42 ± 0.11 ^B^	1.27 ± 0.08 ^B^	-	93.7 ± 1.23 ^A^	0.321 ± 0.040 ^A^	4.61
P0	90.46 ± 0.16 ^a^	0.36 ± 0.17 ^a^	11.89 ± 0.11 ^b^	1.26 ± 0.17 ^a^	0.27 ± 0.17 ^a^	0.339 ± 0.011 ^c^	86.22
P5	90.83 ± 0.45 ^a^	0.74 ± 0.22 ^a^	11.76 ± 0.39 ^b^	1.13 ± 0.19 ^a^	3.14 ± 0.83 ^b^	0.352 ± 0.003 ^cd^	83.23
P10	90.82 ± 0.05 ^a^	0.63 ± 0.05 ^a^	11.47 ± 0.71 ^ab^	1.04 ± 0.16 ^a^	3.94 ± 0.84 ^b^	0.320 ± 0.006 ^d^	82.92
P15	91.51 ± 0.30 ^a^	1.06 ± 0.82 ^b^	11.18 ± 0.64 ^ab^	0.97 ± 0.18 ^a^	6.55 ± 0.41 ^c^	0.298 ± 0.008 ^a^	80.24
P20	92.25 ± 0.49 ^a^	1.41 ± 0.37 ^b^	10.34 ± 0.34 ^a^	0.96 ± 0.18 ^a^	7.05 ± 0.82 ^c^	0.302 ± 0.004 ^ab^	80.24

* CWF—common wheat flour, MOH—micronized oat husk, P0, P5, P10, P15 and P20—pasta with 0, 5, 10, 15 and 20 g/100 g of micronized oat husk, respectively. ** The values designated by the different small letters (a,b,c,d) and big letters (A,B) in the columns of the table are significantly different (α = 0.05).

**Table 2:** The color parameters of raw materials and pasta samples.

Sample	Color Parameters			
	L*	a*	b*	Δ*E*
CWF *	90.76 ± 0.68 ^B^**	2.05 ± 0.07 ^A^	13.79 ± 0.05 ^A^	-
MOH	74.51 ± 0.14 ^A^	8.13 ± 0.07 ^B^	22.66 ± 0.06 ^B^	-
P0	66.48 ± 4.37 ^d^	3.27 ± 0.29 ^a^	13.63 ± 1.04 ^a^	-
P5	64.52 ± 0.63 ^cd^	3.97 ± 0.04 ^a^	14.45 ± 2.48 ^a^	4.70 ± 1.55 ^a^
P10	60.07 ± 1.72 ^c^	5.03 ± 0.38 ^b^	15.00 ± 0.15 ^a^	7.38 ± 3.29 ^b^
P15	55.54 ± 2.24 ^b^	5.62 ± 0.42 ^bc^	15.90 ± 1.10 ^a^	11.29 ± 5.49 ^bc^
P20	48.66 ± 1.75 ^a^	6.09 ± 0.04 ^c^	16.67 ± 0.59 ^a^	18.02 ± 4.63 ^c^
CP0	67.26 ± 0.89 ^b^	2.02 ± 0.18 ^a^	16.51 ± 0.05 ^a^	-
CP5	66.45 ± 1.39 ^b^	5.38 ± 0.07 ^b^	20.44 ± 1.61 ^ab^	5.34 ± 1.33 ^a^
CP10	62.97 ± 2.28 ^b^	7.59 ± 0.76 ^c^	22.02 ± 3.30 ^b^	9.20 ± 3.75 ^b^
CP15	63.33 ± 3.17 ^b^	8.15 ± 0.55 ^cd^	23.69 ± 0.29 ^b^	10.48 ± 1.24 ^bc^
CP20	53.17 ± 2.08 ^a^	9.62 ± 1.21 ^d^	22.04 ± 2.27 ^b^	16.96 ± 3.77 ^c^

* CWF—common wheat flour, MOH—micronized oat husk, P0, P5, P10, P15 and P20—uncooked pasta with 0, 5, 10, 15 and 20 g/100 g of micronized oat husk, respectively, CP0, CP5, CP10, CP5 and CP20—cooked pasta with 0, 5, 10, 15 and 20 g/100 g of micronized oat husk, respectively. L*—lightness, a*—redness, b*—yellowness, Δ*E*—total color difference, ** The values designated by the different small letters (a,b,c,d) and big letters (A,B) in the columns of the table are significantly different, *n* = 3, (*p* < 0.05).

**Table 3:** Cooking properties of pasta.

Sample	Parameters		
	OCT (min)	WI (kg CP/kg P)	CL (g/100 g of pasta)
CP0 *	10.50 ± 0.26 ^a^**	2.28 ± 0.02 ^c^	4.62 ± 0.02 ^a^
CP5	10.90 ± 0.22 ^ab^	2.26 ± 0.03 ^c^	5.46 ± 0.03 ^b^
CP10	11.50 ± 0.23 ^b^	2.22 ± 0.03 ^b^	5.49 ± 0.03 ^b^
CP15	12.10 ± 0.13 ^c^	2.14 ± 0.02 ^ab^	5.57 ± 0.03 ^b^
CP20	12.60 ± 0.21 ^c^	2.10 ± 0.01 ^a^	7.52 ± 0.01 ^c^

* CP0, CP5, CP10, CP5 and CP20—cooked pasta with 0, 5, 10, 15 and 20 g/100 g of micronized oat husk, respectively. OCT—optimum cooking time (min), WI—weight increase index (kg·kg^−1^), CL—cooking loss (kg·kg^−1^), CP—cooked pasta, P—uncooked pasta. ** Mean ± SD, *n* = 3. Values followed by the same letter (a,b,c) in the same rows are not significantly different (*p* < 0.05).

**Table 4:** Textural properties of pasta.

Sample	Parameters	
	Deformation (mm)	Stretching Force (N)
CP0 *	7.04 ± 3.15 ^b^**	1.06 ± 0.07 ^b^
CP5	5.54 ± 1.34 ^ab^	0.83 ± 0.10 ^a^
CP10	4.98 ± 1.79 ^ab^	0.75 ± 0.13 ^a^
CP15	3.94 ± 1.13 ^ab^	0.74 ± 0.06 ^a^
CP20	2.82 ± 0.69 ^a^	0.73 ± 0.06 ^a^

* CP0, CP5, CP10, CP5 and CP20—cooked pasta with 0, 5, 10, 15 and 20 g/100 g of micronized oat husk, respectively. ** Mean ± SD, *n* = 3. Values followed by the same letter (a,b) in the same rows are not significantly different (*p* < 0.05).

**Table 5:** TPC and AA of raw materials and cooked pasta.

Sample	Parameters		
	TPC (mg GEA/g d. m.)	ABTS EC_50_ (mg d. m./mL)	DPPH EC_50_ (mg d. m./mL)
CWF *	256.87 ± 10.06 ^A^**	242.69 ± 12.07 ^A^	355.12 ± 2.71 ^A^
MOH	628.32 ± 12.35 ^B^	35.62 ± 1.06 ^B^	55.22 ± 2.73 ^B^
CP0	247.59 ± 1.12 ^a^	256.53 ± 2.01 ^e^	364.04 ± 10.99 ^e^
CP5	263.24 ± 13.87 ^ab^	186.04 ± 5.10 ^d^	282.37 ± 3.05 ^d^
CP10	279.61 ± 3.40 ^ab^	154.93 ± 7.62 ^c^	252.40 ± 2.20 ^c^
CP15	291.37 ± 8.99 ^b^	136.85 ± 8.92 ^b^	206.23 ± 4.29 ^b^
CP20	350.20 ± 24.49 ^c^	101.17 ± 1.56 ^a^	162.02 ± 8.45 ^a^

* CWF—common wheat flour, MOH—micronized oat husk, CP0, CP5, CP10, CP5 and CP20—cooked pasta with 0, 5, 10, 15 and 20 g/100 g of micronized oat husk, respectively. ** Mean ± SD, *n* = 3, separate statistical analyses of TPC, ABTS and DPPH were performed; different small letters (a,b,c,d,e) and big letters (A,B) in the columns of the table mean significant differences between means (*p* < 0.05).

**Table 6:** Flavonoid aglycones and glycosides content.

Sample	Flavonoid Aglycones (µg/g d.m.)		Flavonoid Glycosides (µg/g d.m.)	
	Luteolina	Apigenina	Luteolin-7-*O*-glucoside (Luteoloside)	Apigenin-6-C-glucoside (Isovitexin)/ Apigenin-8-C-glucoside (Apigenin-6-C-glucoside (Vitexin)
P0 *	trace	trace	trace	trace
P5	trace	trace	nd	trace
P10	trace	trace	nd	trace
P15	trace	trace	nd	19.75 ± 0.21 ^a^**
P20	trace	trace	nd	28.40 ± 0.92 ^a^

* P0, P5, P10, P15 and P20—pasta with 0, 5, 10, 15 and 20 g/100 g of micronized oat husk, respectively. * Average (*n* = 3), trace—trace amounts, nd—not detected. ** Mean ± SD, *n* = 3. Values followed by the same letter (a) in the same rows are not significantly different (*p* < 0.05).

**Table 7:** Phenolic acids contents of pasta supplemented with MOH.

Phenolic Acids (µg/g d.m.)	Sample				
	P0 *	P5	P10	P15	P20
** 3-*O*-caffeoylquinic acid (neochlorogenic acid)	nd	nd	trace	trace	trace
*** 5-caffeoylquinic acid (chlorogenic acid)	trace	trace	trace	trace	4.35 ± 0.01 ^a^**
4-Hydroxybenzoic acid	trace	trace	trace	trace	trace
vanilic acid	trace	trace	trace	8.25 ± 0.50 ^a^	19.33 ± 0.04 ^ab^
syringic acid	nd	nd	trace	15.95 ± 0.07 ^a^	31.85 ± 0.71 ^b^
4-Hydroxycinnamic acid (p-coumaric acid)	trace	54.00 ± 4.21 ^a^	149.75 ± 1.06 ^a^	266.75 ± 3.18 ^b^	292.00 ± 0.71 ^c^
ferulic acid	643.50 ± 36.74 ^a^	1025.50 ± 9.19 ^b^	1227.50 ± 17.68 ^b^	1522.50 ± 31.82 ^c^	1517.50 ± 38.89 ^d^
salicylic acid	trace	trace	trace	trace	trace

* P0, P5, P10, P15 and P20—pasta with 0, 5, 10, 15 and 20 g/100 g of micronized oat husk, respectively. Average (n = 3), ** according to fragmentation 1, *** according to fragmentation 2, trace—trace amounts, nd—not detected. ** Mean ± SD, *n* = 3. Values followed by the same letter (a,b,c,d) in the same rows are not significantly different (*p* < 0.05).

**Table 8:** Sensory characteristics of cooked pasta fortified MOH.

Sample	Sensory Attribute					
	Appearance	Color	Smell	Taste	Texture	Overall
CP0 *	2.67 ± 0.58 ^a^**	2.33 ± 1.15 ^a^	6.33 ± 0.58 ^a^	5.67 ± 0.58 ^b^	6.00 ± 0.00 ^b^	4.67 ± 0.58 ^ab^
CP5	4.33 ± 1.15 ^ab^	4.33 ± 0.58 ^ab^	6.00 ± 0.00 ^a^	5.67 ± 0.58 ^b^	5.67 ± 0.58 ^b^	6.00 ± 0.02 ^c^
CP10	6.33 ± 0.58 ^c^	5.67 ± 1.15 ^b^	5.67 ± 0.58 ^a^	5.00 ± 0.03 ^ab^	4.67 ± 0.58 ^ab^	6.33 ± 0.58 ^c^
CP15	6.00 ± 0.02 ^bc^	6.00 ± 0.02 ^b^	5.67 ± 0.58 ^a^	5.33 ± 0.58 ^b^	4.67 ± 0.58 ^ab^	5.00 ± 0.02 ^b^
CP20	6.33 ± 0.58 ^c^	6.00 ± 0.03 ^b^	5.67 ± 0.58 ^a^	4.00 ± 0.02 ^a^	3.67 ± 0.58 ^a^	4.00 ± 0.03 ^a^

* CP0, CP5, CP10, CP15 and CP20—cooked pasta with 0, 5, 10, 15 and 20 g/100 g of micronized oat husk, respectively. Acceptability on a 7-point hedonic scale. ** Mean ± SD, *n* = 3. Values followed by the same letter (a,b,c) in the same rows are not significantly different (*p* < 0.05).

## Data Availability

The data presented in this study are available on request from the corresponding author.

## Sample Availability

Samples of the compounds are available from the authors.
